# eDNA-based monitoring of *Batrachochytrium dendrobatidis* and *Batrachochytrium salamandrivorans* with ddPCR in Luxembourg ponds: taking signals below the Limit of Detection (LOD) into account

**DOI:** 10.1186/s12862-023-02189-9

**Published:** 2024-01-05

**Authors:** David Porco, Chanistya Ayu Purnomo, Liza Glesener, Roland Proess, Stéphanie Lippert, Kevin Jans, Guy Colling, Simone Schneider, Raf Stassen, Alain C. Frantz

**Affiliations:** 1 Musée national d’histoire naturelle du Luxembourg, 25, rue Münster, Luxembourg, L-2160 Luxembourg; 2 Fondation Faune Flore, 24, rue Münster, Luxembourg, L-2160 Luxembourg; 3 Naturschutzsyndikat SICONA, 12, rue de Capellen, L-8393 Olm, Luxembourg, Luxembourg; 4 Umweltplanungsbüro Ecotop, 45, Schlassuecht, L-7435 Hollenfels, Luxembourg, Luxembourg; 5 Natur&ëmwelt Fondation Hëllef fir d’Natur, 5, Route de Luxembourg, L-1899, Kockelscheuer, Luxembourg; 6 Biota.lu, 9a, Rue Principale, L-6990, Hostert, Luxembourg

**Keywords:** *Batrachochytrium dendrobatidis*, *Batrachochytrium salamandrivorans*, eDNA, Below-LOD signal, ddPCR, Pathogens, Monitoring, Luxembourg

## Abstract

**Background:**

*Batrachochytrium dendrobatidis* (*Bd*) and *Batrachochytrium salamandrivorans* (*Bsal*) are two pathogenic fungi that are a significant threat to amphibian communities worldwide. European populations are strongly impacted and the monitoring of the presence and spread of these pathogens is crucial for efficient decision-making in conservation management.

**Results:**

Here we proposed an environmental DNA (eDNA) monitoring of these two pathogenic agents through droplet digital PCR (ddPCR) based on water samples from 24 ponds in Luxembourg. In addition, amphibians were swabbed in eight of the targeted ponds in order to compare the two approaches at site-level detection. This study allowed the development of a new method taking below-Limit of Detection (LOD) results into account thanks to the statistical comparison of the frequencies of false positives in no template controls (NTC) and below-LOD results in technical replicates. In the eDNA-based approach, the use of this method led to an increase in *Bd* and *Bsal* detection of 28 and 50% respectively. In swabbing, this resulted in 8% more positive results for *Bd*. In some samples, the use of technical replicates allowed to recover above-LOD signals and increase *Bd* detection by 35 and 33% respectively for eDNA and swabbing, and *Bsal* detection by 25% for eDNA.

**Conclusions:**

These results confirmed the usefulness of technical replicates to overcome high levels of stochasticity in very low concentration samples even for a highly sensitive technique such as ddPCR. In addition, it showed that below-LOD signals could be consistently recovered and the corresponding amplification events assigned either to positive or negative detection via the method developed here. This methodology might be particularly worth pursuing in pathogenic agents’ detection as false negatives could have important adverse consequences. In total, 15 ponds were found positive for *Bd* and four for *Bsal*. This study reports the first record of *Bsal* in Luxembourg.

**Supplementary Information:**

The online version contains supplementary material available at 10.1186/s12862-023-02189-9.

## Background

Two chytrid fungi, *Batrachochytrium dendrobatidis* (*Bd*) and *Batrachochytrium salamandrivorans* (*Bsal*), possibly originating from Asia and introduced in other continents through pet trade, were identified as major drivers responsible for the global decline of amphibians observed over the last decades [1–4]. The systematic monitoring of these skin pathogenic fungi lethal to amphibians is crucial as it allows investigating both the local and general virulence and dispersal patterns, while considering co-factors such as environmental conditions (e.g. temperature, humidity, cover) or human activities (e.g. trade entry points, pet trade, nitrous fertilization) [4, 5]. Moreover, accumulating and aggregating monitoring data could help in the fine-tuning of local niche models [6] and lead to enhanced safety in conservation actions such as reintroduction and translocation projects [7].

Initially, the detection of these chytrid fungi relied on histopathology [8] and immune-histochemical assays [9, 10]. In recent surveys, the detection shifted to molecular-based methods with several qPCR detection assays designed to detect the DNA of both chytrid fungi based on specific primers and probes [11–13]. These molecular methods enabled the detection of the pathogens at any stage of their life cycle and were first applied to DNA extracts from toe clips and skin swabbing [11, 12]. In the last decades, the development of approaches based on environmental DNA (eDNA) allowed the recovery of organisms’ DNA from environmental samples such as air, soil or water [14]. The *Bd*/*Bsal* detection benefited from these advances and an increasing number of monitoring studies were based on eDNA extracted from water samples [15–22].

Droplet digital PCR (ddPCR) is increasingly being used in the field of eDNA. Through emulsion partitioning, ddPCR produces thousands of independent PCR reactions allowing for high-sensitivity detection and absolute quantification of template concentration in samples [23, 24]. Partially overcoming inhibition effects and known to outcompete qPCR in low eDNA concentrations, this technique is gaining popularity in the field of eDNA-based detection (e.g. [25–30]). However, the use of ddPCR for the detection of the chytrid pathogenic fungi has remained limited and indirect to date. For instance, it has been employed to evaluate, in *Bd* positive samples, the copy number in different strains [31] or to quantify *Bd*-positive samples in a qPCR survey [32]. However, given its high sensitivity, the direct use of ddPCR has the potential to significantly increase the efficiency of *Bd* and *Bsal* detection.

In eDNA-based detection, the very low concentrations of target DNA often result in signals that are difficult to delineate from the background noise associated with the methodology. These very low template concentrations may lead to the generation of signals below the Limit of Detection (LOD i.e. the lowest concentration of target DNA that can be detected with sufficient confidence e.g. [33]), and also increase the stochasticity in amplifications [34]. Thus, below-LOD signals could originate from the actual amplification of the target DNA and could be worth sorting apart from mere background noise. Indeed, in either analytical chemistry or eDNA-based detection, below-LOD values were often proven to convey valuable information [35–40]. However, in eDNA studies, LOD can be used as a threshold below which amplification events are disregarded (e.g. [41–43]). Discarding these signals could bias detection and produce false negatives, which can be particularly perilous when the object of detection is a pathogenic agent. For this type of target, decreasing false negatives should be highly promoted [37, 44], as non-detection could have important impacts. The fact that below-LOD results could originate from the actual presence of the pathogen was already suggested in a previous *Bsal* swabbing survey, but these could still not be considered genuine positives [45].

The presence of *Bd* has been confirmed in Luxembourg in 2009 [46, 47] though the zoospore load was generally lower than the one observed during severe chytridiomycosis outbreaks [47]. Concerning *Bsal*, despite its presence in the border region of neighbouring countries, it was not detected in Luxembourg [48]. Concomitantly, the decline of amphibian populations in Luxembourg in recent decades has prompted various measures such as legal protection, road-crossing structures, habitat improvement, and breeding pond creation or restoration [49–52]. Translocation or reintroduction programs have also been employed to protect or restore the populations of some species [51]. However, given the risk posed by *Bd* and the high rate of spread of *Bsal* [53], such initiatives, without adequate monitoring, could result in the transfer of naïve amphibians into contaminated ponds or conversely the introduction of potential pathogen reservoir specimens into ponds originally devoid of pathogens.

In the present study, ddPCR was used for the detection of the fungal pathogens *Bd* and *Bsal* in both eDNA-based and swab-based monitoring of 24 and 8 ponds respectively in Luxembourg. This survey allowed to further investigate the use of below-LOD amplification events as valid detection events through a comparison with the background noise generated from the methodology and assays.

## Results

None of the negative controls exhibited amplification, thus positives can soundly be assumed to originate from genuine template amplification.

### *Bd*

The investigation concerning the background artefact for the *Bd* assay yielded two amplifications out of 190 NTC. Limit of Blank (LOB) was 0.48 cp/μl (copies per μl), the derived LOD 2.15 cp/μl and Limit of Quantification (LOQ) 3.54 cp/μl. Specific correction factors for the delineation of positive amplification events were calculated for each pond sampled in order to fit the impact of inhibition on their fluorescence level (Table S1).

**Fig. 1:**
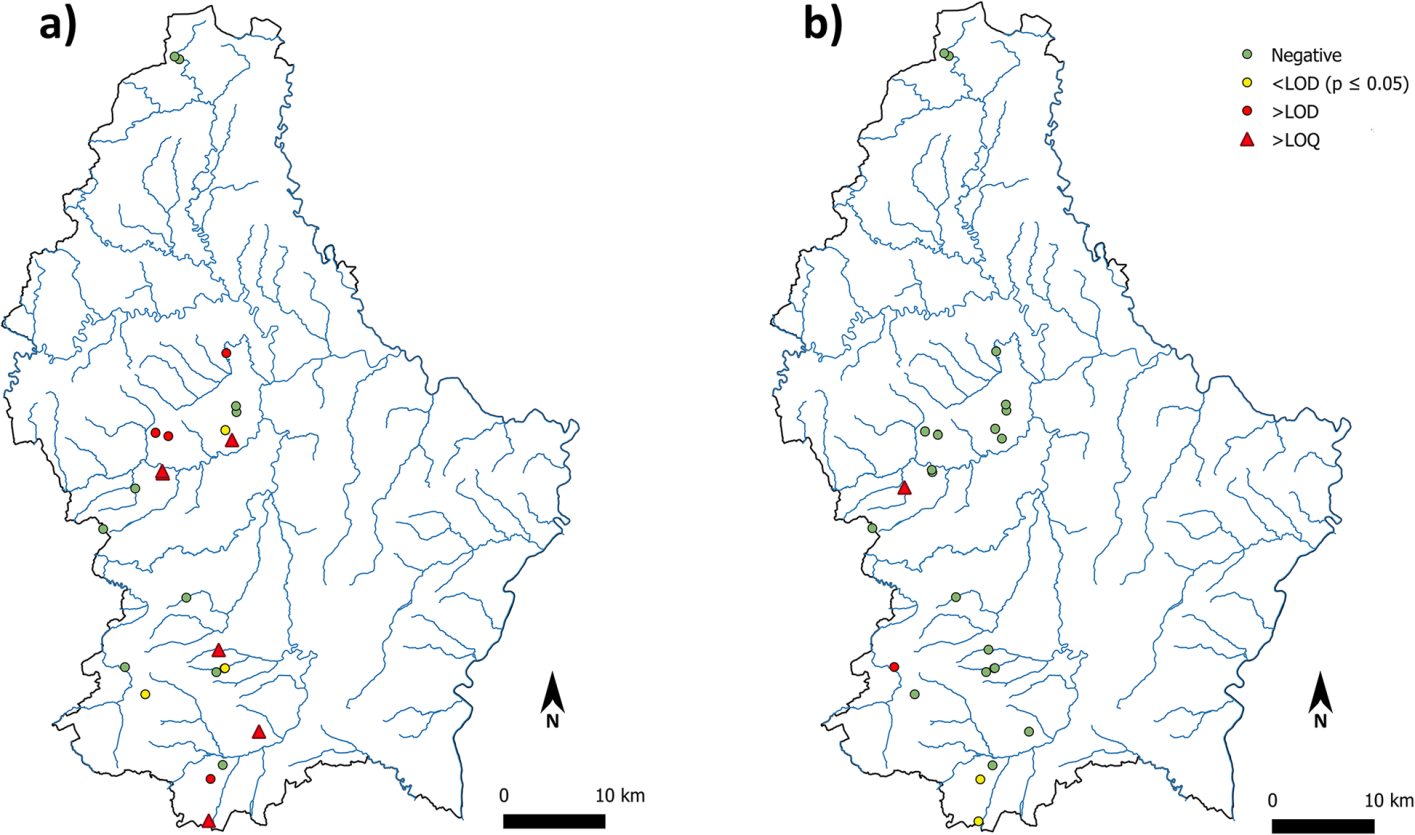
Map of the detection results for **a**) *Bd* and **b**) *Bsal*. Green dots = negatives; yellow dots = positives < LOD (frequency of < LOD events significantly higher than NTC false positives frequency); red dots = positive > LOD; red triangle = positives > LOQ. This map was generated using QGIS 3.2.1 [54]

For eDNA amplifications, 14 out of the 24 sites sampled were flagged as *Bd*-positive (Fig. 1a, Table 1): ten with concentrations above LOD and four with measurements below LOD but for which the frequency of amplification events was found statistically different from artefacts frequency in NTC (Table 1). Globally, the concentrations measured ranged from 0 to 113.73 cp/μl (Table S2).

**Table 1:** Results of *Bd* detection for eDNA and swabbing approaches. Chi^2^ test = Pearson’s Chi-squared test to compare the frequencies of artefacts in NTC and below-LOD amplifications

Sites	eDNA													
	First pass results			Replicates repetions			Positive replicates <LOD repetitions			Total				
	>LOD	<LOD	Total	>LOD	<LOD	Total	>LOD	<LOD	Total	>LOD	<LOD	Total	Chi^2^ test	Site status
H-BEC85	0	0	0											NEG
H-Bert47	0	0	0											NEG
H-Bert49	0	1	1/10	0	2	2/20	0	1	1/13	0	4	4/43	*p* = 0.01349	POS
H-Bert50	0	1	1/10	0	2	2/20	0	0	0/13	0	3	3/43	*p* = 0.03898	POS
H-BETB03	1	0	1/10											POS
H-BETM03	0	4	4/10	2	5	7/20	15	14	29/52			40/82		POS
H-BI147	0	1	1/10	0	1	1/20	0	2	2/13	0	4	4/43	*p* = 0.01349	POS
H-KE77	0	0	0											NEG
T-BE24	0	0	0											NEG
T-Bert39	4	4	8/10											POS
T-BI09	10	0	10/10											POS
T-BK93	0	0	0											NEG
T-FE49	0	1	1/10	0	2	2/20	1	4	5/13	1	7	8/43		POS
T-KA41	1	1	2/10											POS
T-KABO2	0	1	1/10	0	7	7/20	0	4	4/13	0	12	12/43	*p* = 4.998e-04	POS
T-KAE45	0	0	0											NEG
TR-BK15	0	0	0											NEG
T-RED09	0	0	0											NEG
T-RU27	0	1	1/10	1	1	2/20	0	2	2/13	1	4	5/43		POS
T-US03	0	1	1/10	4	5	9/20	9	3	11/12	13	9	22/42		POS
T-US04	0	1	1/10	2	1	3/20	0	2	2/13	2	4	6/43		POS
T-US042	1	1	2/10											POS
T-X01	0	0	0											NEG
T-X02	0	0	0											NEG

Swabbing											
	First pass results						Positive sample < LOD repetitions				
N	>LOD	Specimen number	ID	<LOD	Specimen number	ID	>LOD	<LOD	Total	Chi^2^ test	Site status
29	0			0							NEG
30	1	HF-Bert47–27	*Hyla arborea*	1	HF-Bert47–29	*Hyla arborea*	9	11	20/20		POS
30	3	HF-Bert49–26/28/29	*Hyla arborea*	1	HF-Bert49–27	*Hyla arborea*	4	9	14/20		POS
30	0			0							NEG
30	1	HF-BETB03–1	*Pelophylax* sp.	0							POS
30	2	HF-BETM03–9/11	*Pelophylax* sp.	1	HF-BETM03–5	*Pelophylax* sp.	1	7	9/20		POS
				1	HF-BETM03–7	*Pelophylax* sp.	2	4	7/20		POS
30	0			1	HF-BI147–13	*Ichthyosaura alpestris*	0	1	2/20	*p* = 0.04648	POS
30				1	TRF-BK15–11		0	0	1/20	NS	NEG
				1	TRF-BK15–26		0	0	1/20	NS	NEG

N = number of specimens swabbed. When only < LOD signals were obtained in the first pass, the total number of replicates per site was obtained through the amplification of the 10 field replicates, the two repetitions of these along with the 12–13 repetitions for the field replicates that yielded < LOD signal

Among the 14 amphibian specimens found positive with swabbing, seven yielded signals above LOD and seven below LOD in the first pass (Table 1). After the amplification of additional technical replicates in below-LOD signals, four specimen extracts yielded signals above LOD and one a below-LOD signal (which was found positive when statistically compared to NTC background noise). Two specimen extracts produced no additional signals (Table 1). In total, four out of the eight sites surveyed in swabbing yielded a positive detection for Bd. Among all samples, concentrations ranged from 0 to 409.26 cp/μl (Table S2).

Out of 12 positive specimens, 11 were anurans (*Hyla arborea* and *Pelophylax* sp.). The remaining positive signal (below LOD, but yielding significantly more amplification events than NTC) was produced from the Urodela species *Ichthyosaura alpestris*. Thus, in spite of a strong taxonomic sampling bias, with most of the specimens sampled belonging to Urodela (Table S3–88% urodelans and 12% anurans), *Bd* detection was mostly achieved in Anura (Table S3–39.3% in anurans and 0.7% in urodelans).

Six out of the eight sites where amphibian specimens were swabbed were also found positive with the eDNA approach (Table 1). The two remaining *Bd*-positive sites showed disagreement between eDNA and swabbing approaches: the site H-Bert47, where eDNA yielded no amplification, and the site H-Bert50, where none of the extracts from specimen swabbing produced a signal (Table 1).

### *Bsal*

The background artefact assessment for this assay yielded two amplifications out of 182 NTC samples. LOB was 0.51 cp/μl, derived LOD 2.18 cp/μl and LOQ 3.35 cp/μl. Specific correction factors for the delineation of positive amplification events were calculated for each pond sampled (Table S1).

In eDNA-based detection, four ponds were found positive for *Bsal*, two above LOD and two below LOD (but statistically different from NTC artefact background noise) (Fig. 1b, Table 2). Globally, concentrations ranged from 0 to 7.04 cp/μl (Table S2).

**Table 2:** Results of *Bsal* detection for eDNA and swabbing approaches. Chi^2^ test = Pearson’s Chi-squared test to compare the frequencies of artefacts in NTC and below-LOD amplifications

Sites	eDNA													
	First pass results			Replicates repetions			Positive replicates repetitions				Total			
	>LOD	<LOD	Total	>LOD	<LOD	Total	>LOD	<LOD	Total	>LOD	<LOD	Total	Chi^2^ test	Site status
H-BEC85	0	0	0											NEG
H-Bert47	0	1	1/10	0	0	0/20	0	0	0/13	0	1	1/43	NS	NEG
H-Bert49	0	0	0											NEG
H-Bert50	0	0	0											NEG
H-BETB03	0	1	1/10	0	0	0/20	0	0	0/13	0	1	1/43	NS	NEG
H-BETM03	0	0	0											NEG
H-BI147	0	0	0											NEG
H-KE77	0	0	0											NEG
T-BE24	0	0	0											NEG
T-Bert39	0	0	0											NEG
T-BI09	0	0	0											NEG
T-BK93	0	0	0											NEG
T-FE49	0	0	0											NEG
T-KA41	0	1	1/10	0	2	2/20	0	0	0/13	0	3	3/43	*p* = 0.04698	POS
T-KABO2	0	0	0											NEG
T-KAE45	1	0	1/10											POS
TR-BK15	0	0	0											NEG
T-RED09	0	1	1/10	1	1	2/20	0	0	0/13	1	2	3/43		POS
T-RU27	0	1	1/10	0	2	2/20	0	0	0/13	0	3	3/43	p = 0.04698	POS
T-US03	0	0	0											NEG
T-US04	0	1	1/10	0	0	0/20	0	0	0/13	0	1	1/43	NS	NEG
T-US042	0	0	0											NEG
T-X01	0	0	0											NEG
T-X02	0	0	0											NEG

Swabbing							
	First pass results		Positive sample < LOD repetitions				
N	>LOD	<LOD	>LOD	<LOD	Total	Chi^2^ test	Site status
29	0	1	0	1	1/20	NS	NEG
30	0	0					NEG
30	0	0					NEG
30	0	0					NEG
30	0	0					NEG
30	0	0					NEG
30	0	0					NEG
30	0	0					NEG

N = number of specimens swabbed. When only < LOD signals were obtained in the first pass, the total number of replicates per site was obtained through the amplification of the 10 field replicates, the two repetitions of these along with the 12–13 repetitions for the field replicates that yielded < LOD signal

None of the extracts from specimen swabbing yielded a detection for *Bsal*. However, as there was no overlap between the eDNA positive sites and the swabbing experiment no comparison or confirmation could be gained via the swabbing approach (Table 2).

## Discussion

### *Bd* detection

**Fig. 2:**
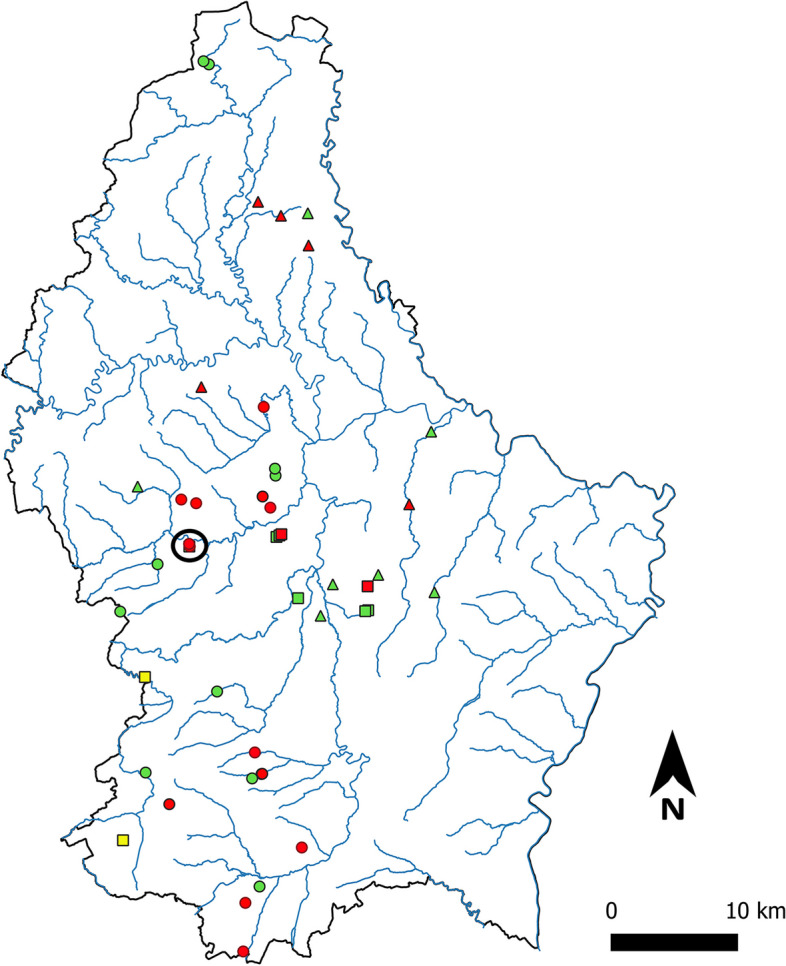
Map summarizing *Bd* detection results for the sites sampled in [46] (green squares = negatives; red squares = positives), in [47] (green triangles = negatives; red triangles = positives) and in this study (green dots = negatives; red dots = positives). The black circle highlights the Useldange site that was tested negative in 2008 [46] and positive in this study. This map was generated using QGIS 3.2.1 [54]

*Bd* was successfully detected in 15 out of the 24 sites sampled either through the eDNA, the swabbing approach or both (Fig. 1a). Two previous studies based on specimen swabbing already detected *Bd* in Luxembourg. The first one detected the pathogenic fungus in the central part of the country in two out of eight sites surveyed; in addition, two other sites provided results below the LOD in southwestern Luxembourg and were considered as ‘possible’ positive sites [46] (Fig. 2). These uncertain detection events could have benefited from a higher repetition level and a comparison with NTC artefactual frequency as implemented here. The second study, undertaken a few years later, detected *Bd* in five out of 12 sites surveyed, mainly in the northern and eastern part of the country [47] (Fig. 2). Altogether the results of the three studies show that *Bd* is well implanted in Luxembourg and likely spreading: one locality surveyed and found negative in 2008 [46], Useldange, was found positive in the present study (Fig. 2).

While *Bd* infection is often lethal in anurans [3], urodelans usually recover from infection and were thus flagged as potential reservoirs [55]. However, low *Bd* prevalence was found in wild urodelans populations [56] and some species were proven resistant possibly due to skin defenses [57]. Thus, urodelans could actually be less susceptible to act as pathogen reservoirs as previously thought. This could explain the unbalanced detection ratio found in this study, with a much higher rate of positives in anurans (Table S3–39.3% in anurans and 0.7% in urodelans) despite the lower sampled number of specimens for this group (Table S3–88% urodelans and 12% anurans). It is notable that in the course of the swabbing not a single animal with visible signs of chytridiomycosis could be detected.

### *Bsal* detection

*Bsal* was detected in four of the 24 sites sampled using eDNA (Fig. 1b). Although this pathogen was already detected in two of the neighboring countries of Luxembourg, Belgium and Germany [6, 58], this is the first detection event of *Bsal* for the country itself. The positive sites were located in the western part of the country, which could mean that *Bsal* may have spread already across several regions of Luxembourg (but see below in ‘Potential biological significance of low-level signals’ concerning the pathogen vectors). While this finding should be further confirmed through methods other than eDNA alone, such as swabbing or observation of chytridiomycosis symptoms in wild specimens, it is an early warning for Luxembourg but also for other European countries as it suggests that *Bsal* is currently spreading.

### Retrieving positive signals below-LOD

In order to achieve a higher degree of confidence in below-LOD amplification events, background noise signal was defined in each assay through the amplification of numerous NTC replicates (in ddPCR, such a background noise i.e. positive amplification artefacts in NTC was previously described in several studies e.g. [59–61]). This allowed to assess the background signal inherent to each ddPCR assay, so it could be compared statistically with repetitions of samples that yielded below-LOD signals. Those samples had to be replicated sufficiently to make statistical comparisons possible. This increase in technical replicates in very low template samples was already known to help counter the stochasticity and the resulting sampling effect [34]. Here, the comparison of their amplifications results to background noise was added to further ascertain the detection.

**Fig. 3:**
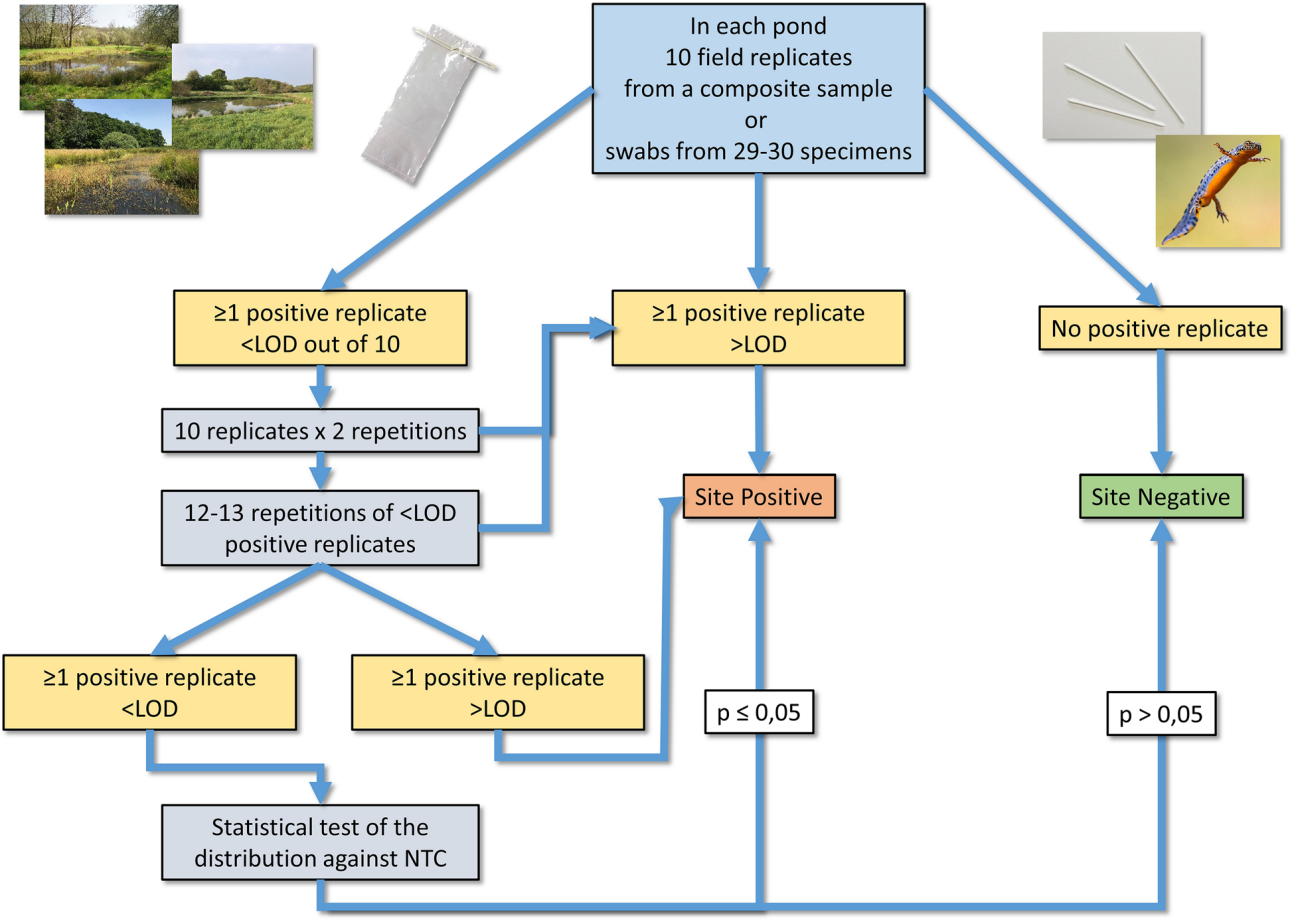
Schematic representation of *Bd*/*Bsal* detection process and decision-making

In this study, for the sites that yielded below-LOD signals in the first pass (Fig. 3), the application of this approach allowed to recover signals higher than the LOD but also to gain positive detections from the comparison of below-LOD signals with the NTC background. This yielded additional detection events originating from very low template concentrations possibly produced by low-density fungus populations. Using this approach, and in comparison to considering below-LOD signals as negatives, the eDNA-based detection increased by 28 and 50% respectively for *Bd* and *Bsal*. In swabbing, it brought 8% more positive results for *Bd*. It is also worth noting that the repetition in itself allowed to gain higher level signals (i.e. above LOD) that also significantly increased the number of detection events: in *Bd*, by 35% for eDNA and 33% for swabbing and 25% for *Bsal* eDNA detection. This highlights the risk of false negatives due to high levels of stochasticity in very low concentration samples and thus the importance of repetitions to retrieve the signal even for a highly sensitive technique such as ddPCR.

Although this approach is replicate-intensive, it helps to objectively formalize the recovery of below-LOD amplifications in the detection process. However, it is worth noting that, due to low concentration template amplification stochasticity, the two steps of technical repetition (i.e. the two repetitions of all field replicates and the 12–13 repetitions of < LOD signals from the original amplification of the 10 field replicates, Fig. 3) should be operated separately: if the first pass processed yields any >LOD signal, the site could be considered as positive and the second part of the repetition saved. The H-BETM03 site exemplifies the utility of implementing the repetition amplification through several steps as four field replicates yielded < LOD signals thus increasing accordingly the replication number when the two repetitions of the field replicates sufficed to obtain >LOD signals and thus confirm *Bd* detection (Table 1).

If several scenarios concerning target concentration along with an increasing number of < LOD signals in the ten initial amplifications from the field replicates are to be taken into account, the best strategy to limit the number of replicates to be processed in order to achieve detection, beyond one < LOD replicate, would be to go for two repetitions of all field replicates (Table S4). This is confirmed by the results obtained in several sites (H-BETM03, T-RU27, T-US03 and T-US04 for *Bd* and T-RED09 for *Bsal* - Tables 1 and 2) where > LOD signals were obtained from repetitions for samples that produced only a single < LOD signal in the first place.

This method might be worth pursuing especially in pathogen detection where false negatives can have highly detrimental consequences. Applying it on a routine basis could allow for a higher confidence level in both decision-making and management.

### Potential biological significance of low-level signals

Low-level signals might originate from biotic vectors with low vectorial capacity such as waterfowl transporting encysted spores on the scales of their feet [62–64] or even abiotic events, such as fog and rain, that could also be vectors for viable spores [65]. Their persistent free-living stage can last several years, thus allowing a considerable time lag between spore arrival and the presence of target or reservoir species [17, 66]. As such, even low frequency events that convey but a few viable spores could, in time, contribute to the actual colonization of ponds. This could explain how ponds might be infected over long distances as the pathogens could benefit from the broad dispersion range of waterfowls and/or abiotic vectors.

Thus, low-level detection signals could be worth documenting as they might indicate local introduction events, even if not successful (i.e. signals could originate from decaying spores material which failed to survive [67]) or the presence of a very small pioneer population. Such early warning signals could flag sites for further scrutiny in future management plans.

### Disagreement between eDNA and swabbing results

A disagreement was found between eDNA and swabbing at the site H-Bert47, with no amplification from eDNA (the additional processing of two more technical replicates for each field replicate did not yield any amplification) when two of the DNA extracts from swabbing produced several over and below-LOD signals. This inconsistency might relate to one drawback of the sampling technique employed here (i.e. composite sample cf. ‘Methods’ paragraph), which was designed to produce dependent replicates that could be pooled for statistical tests. Indeed, this sampling design can produce high levels of dilution: one positive 1 L sample could be mixed with nine negative ones and as the ten 0.5 L replicates are sampled from the composite sample, this could lead to a drastic increase of both stochasticity and sampling bias. In turn, this could jeopardize further detection from the corresponding pool of replicates. Hence, the independent field replicates approach might prove more efficient than the composite sample strategy adopted in this study, as no signal dilution would hamper amplification. However, independent field replicates would require a higher number of total replicates for the statistical comparison of below-LOD signals with artefacts from NTC as technical replicates could not be pooled per site.

Concerning the site H-Bert50, eDNA yielded a positive result from the statistical comparison with artefactual yield when swabbing did not produce any positive amplification events. This low signal detection suggests a low template concentration and thus a limited presence of *Bd*. This could explain that swabbing missed the signal as only 30 specimens were processed for this pond; a higher number of swabbed specimens might have allowed for the detection of *Bd* on this site through swabbing as well.

Thus, for *Bd* detection at site level, eDNA and swabbing results showed only marginal differences (which might be improved by applying independent sampling for eDNA). However, eDNA monitoring, contrary to swabbing, allows disconnecting the pathogen detection from the presence of amphibian hosts. This can be crucial as chytrid fungi could persist for several years in a pond without either target or reservoir amphibian species [17, 66]. Moreover, the eDNA sampling is less time intensive, thus less costly [20] and is less taxing for the amphibian communities as they are not captured and manipulated as in swabbing. Also, the absence of specimen handling and the shorter sampling time on site in eDNA approach could reduce the risk of accidental cross-contaminations between sites. Nevertheless, beyond site-level detection, and contrary to swabbing, eDNA cannot bring information on the prevalence of the pathogenic agents either at the global amphibian population scale or in the different taxonomic compartments of the communities. Thus, swabbing remains a valuable tool to get crucial fine-scale information on the spreading of *Bd* and *Bsal*.

### Conservation implications

This study shows that *Bd* is well implanted in Luxembourg and likely spreading. The newly reported presence of *Bsal* in the country is alarming as it could negatively affects populations of the fire salamander and the great crested newt [45]. This highlights the continued need for field workers to follow good practices in order to minimize the spread of the pathogenic fungi. A comprehensive national *Bd/Bsal* action plan is currently being developed for Luxembourg. It plans to implement quantitative monitoring of *Bsal* annually at some 20 fire salamander sites near the border with neighboring countries [48] and to perform an eDNA-based monitoring of some 20 different ponds each year. A further implication of these results is the critical need for great caution in amphibian reintroductions and translocations. Given the presence of both pathogenic agents, there are concrete risks of transmitting pathogenic agents either way between destination ponds and transferred animals. Therefore, any reintroduction or translocation initiative should be associated with a pathogen monitoring in both the source and recipient populations and/or ponds. Otherwise, these actions could be counterproductive and spread the pathogens.

## Conclusion

ddPCR allowed to monitor *Bd* and *Bsal* in eDNA samples from pond water and DNA extracts from specimen swabs. The results indicated a strong presence of *Bd* in Luxembourg and allowed to detect *Bsal* for the first time in the western part of the country, implying that the pathogenic agent is gaining ground in Europe.

Dealing with pathogen detection makes the prevention of false negatives crucial as these could interfere with monitoring attempts and thus jeopardize amphibian reintroduction or pathogen eradication attempts. Thus, it is crucial to recover as many detection signals as possible with a certain confidence level. In order to achieve this, a new method was proposed in this study through a thorough examination of the specific artefact levels in NTC for the two assays used and a higher level of replication for below-LOD signal samples. The statistical comparison of the frequencies of both items allowed for an objective and reproducible decision-making on the detection status of the targeted pathogens.

The application of this method allowed to recover a substantial amount of detection events which were likely originating from very low concentration templates. Those could be due to random and discrete propagule introductions through low intensity vectors such as wild birds, fog or rain. Even if some of these events may be non-viable introductions, they are worth documenting when the target monitored is a pathogenic agent such as *Bd* and *Bsal*, as even a mere signal of pathogen introduction is important to consider for management.

## Methods

### Water sampling

**Fig. 4:**
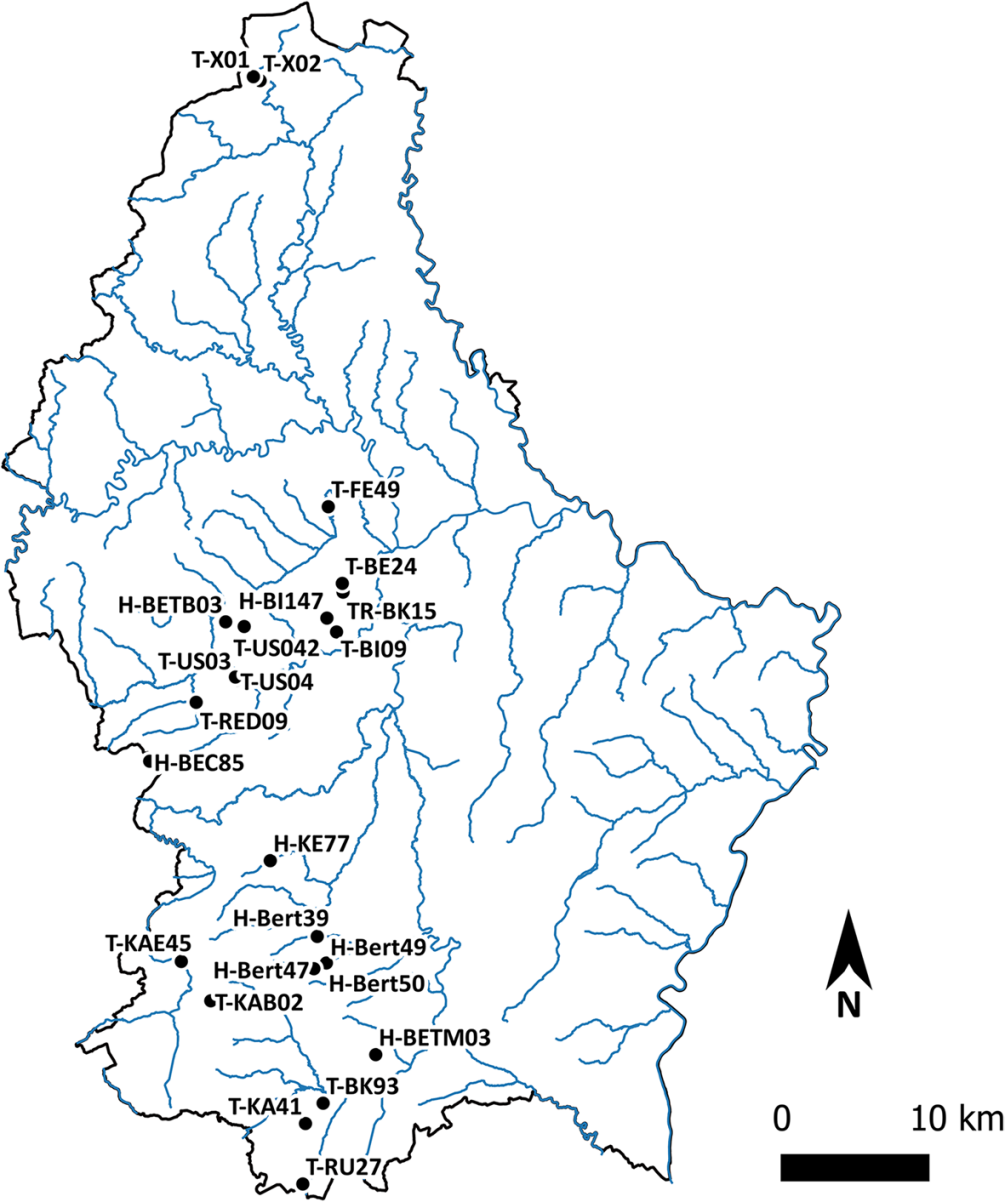
Map of the sites sampled. This map was generated using QGIS 3.2.1 [54]

A network of 24 ponds located in Luxembourg was sampled during mid-April 2022 (Fig. 4, Table 3). At each site, a composite sample was established by mixing 10 1 L samples collected around the whole circumference of the pond. The sampling was conducted at a distance of 15–20 cm from the bank, sampling depth ranged from 5 to 10 cm from the surface. From this composite sample, ten replicates of 500 ml were subsampled using sterile Whirl Pak plastic bags. This sampling protocol was designed to generate dependent replicates for each pond, so these, along with their technical replicates, could be pooled in further statistical analyses.

**Table 3:** Sampling sites (Pond areas were assessed from 2022 aerial photographs and includes the clearly discernible temporarily dry areas (https://www.geoportail.lu/fr/))

Site name	GPS Coordinates	Locality	Swabbing	Pond area in m2
H-BEC85	49.7165265 5.87658436	Beckerich	x	1139
H-Bert47	49.5893123 6.03250296	Bertange-Findelserhaff	x	1134
H-Bert49	49.592832 6.04430518	Bertange-Zéiwelt (Ost)	x	1297
H-Bert50	49.5926783 6.04355868	Bertange-Zéiwelt (West)	x	424
H-BETB03	49.8022853 5.94820655	Bettembourg-Préitzerbierg (Ost)	x	224
H-BETM03	49.5365341 6.09104572	Bettembourg-Léiwesdällchen	x	360
H-BI147	49.8047376 6.0442695	Bissen-Härenhecken	x	967
H-KE77	49.65562812 5.9910636	Kehlen		489
T-BE24	49.8260871 6.05884963	Berg		3433
T-Bert39	49.6091411 6.03564441	Bertange		353
T-BI09	49.7962328 6.05344397	Bissen-am Maart		191
T-BK93	49.5066672 6.04145608	Beckerich-Dréisch		526
T-FE49	49.8731846 6.04541734	Feulen		869
T-KA41	49.4941682 6.02484114	Kayl		196
T-KABO2	49.5694227 5.93516295	Käerjeng-Bommelscheier		611
T-KAE45	49.5935491 5.9071642	Käerjeng-Clemency		241
TR-BK15	49.8207113 6.05955644	Beckerich-Brosiushaff (Plateau)	x	897
T-RED09	49.7527983 5.92042627	Redange		493
T-RU27	49.4570315 6.02243844	Rumeldange		644
T-US03	49.7683986 5.95761803	Useldange-Weiden		408
T-US04	49.766431 5.95836219	Useldange-in den langen Loosen		328
T-US042	49.7994761 5.96580404	Useldange-bei Weweschheck		160
T-X01	50.1373049 5.97315934	Kirchermillen		110
T-X02	50.1348971 5.97958908	Troisvierges		225

Each water sample, which had been stored at 4 °C before processing, was filtered through a 0.45 μm nitrocellulose membrane (Nalgene analytical funnel) using a peristaltic pump (Masterflex L/S Standard Pump Head) connected to a column drill (500 W). The resulting filter was then immediately stored in 800 μl ATL Qiagen lysis buffer at 4 °C in a 2 ml microfuge tube and frozen at − 20 °C back in the laboratory. For most ponds (76%), the filtration could not be performed for the entire 500 ml volume of each replicate due to the presence of high amounts of suspended sediments in the water column (Table S2- range 100–500 ml). For each site, a negative control was used to detect any possible cross-contamination: it consisted of a 500 ml bottle of distilled water that was taken into the field and subjected to the same storage and filtration process as the actual samples.

In order to prevent pathogens spreading between ponds, all the material used for the sampling of a pond was either disposed off (gloves), reused after a wash in 4% hypochlorite and a thorough water rinsing (buckets, bottles) or bleached with 4% hypochlorite (boots, plastic pants).

### Specimen swabbing

For comparison with eDNA results, we conducted amphibian swabbing in eight of the surveyed ponds (Table 3). Up to 30 adult amphibians per pond were caught either in traps (3–4 aquatic funnel traps) or hand-netted for tree frogs. The animals were swabbed according to the following hierarchical order: tree frogs (five individuals in each of the three sites where the species occurred), any other frog or toad, great crested newts, all other newt species. Each animal was swabbed five times over the mouth, the inner thighs (anurans), the base of the tail (urodelans) and the webbing between the toes, resulting in 30 passages per animal. Swab tips were cut and immediately stored at 4 °C in 1.5 ml microfuge tubes containing 180 μl ATL Qiagen lysis buffer and then frozen at − 20 °C back in the laboratory.

To minimize contamination risk during swabbing, two persons were involved in the process: one holding the animal, and the other one swabbing and taking notes (species, sex). Both were wearing nitrile gloves that were discarded between animals. Nets were thoroughly cleaned and disinfected with ethanol (70%) between individuals and ponds. Traps were cleaned and dried for at least 24 hours. Specimen capture and swabbing always took place at a later date than water sampling for eDNA in order to avoid sediment resuspension before water collection. Animals were trapped using permits issued by the Luxembourg Ministry of the Environment, Climate and Biodiversity (References 98551GB/ne, 101,987, 99082GB/ne & 99087GB/ne). All specimens were released after swabbing.

### DNA extraction

#### eDNA

DNA was extracted from the nitrocellulose filter membranes using a Qiagen DNeasy Blood & Tissue Kit with a volume-adapted protocol: after using clean scissors to thoroughly shred the filter directly in its storage tube, 80 μl of Proteinase K were added to the lysis buffer used for filter preservation. After an overnight incubation at 56 °C, 600 μl of the lysis solution were recovered and mixed with 600 μl AL Qiagen lysis buffer (10 minutes incubation at 56 °C) and 600 μl 96% ethanol. After homogenization by mixing, the solution was transferred to Qiagen DNeasy 96 plates. The rest of the protocol followed the manufacturer’s instructions. DNA extracts were eluted in 100 μl of 56 °C-heated AE Qiagen elution buffer. To monitor any potential sample cross-contamination, negative controls (ATL buffer with proteinase K) were extracted alongside each series of samples.

#### Swabs

DNA was extracted from swab tips using a Qiagen DNeasy Blood & Tissue Kit following the manufacturer’s protocol with an overnight lysis at 56 °C. DNA extracts were eluted in 100 μl of 56 °C-heated AE Qiagen elution buffer.

#### ddPCR

The DNA extracts were processed for ddPCR and read on a Bio–Rad QX200 suite according to the manufacturer’s instructions [68]. The ddPCR Supermix for Probes was used along with the specific primer sets previously designed for *Bd* [12] and *Bsal* [13] (with HEX and FAM fluorochromes respectively, quenchers were BHQ1 for both assays). The PCR cycling program followed the manufacturer’s instructions with an annealing temperature of 46 °C for *Bd* and 50 °C for *Bsal* for 50 cycles. The reaction mix was composed of 11 μl ddPCR Supermix for Probes, 760 nM primer and 430 nM probe completed with water to 21 μl and added. It was added with 1 μl template DNA extract for field/technical replicates and 1 μl water for NTC. In addition, several NTC reactions were processed for *Bd* and *Bsal* assays. In each assay, the amplification results from the negative controls and the additional NTC reactions were pooled to assess the frequency of false positives (for a total number of 190 and 182 respectively for *Bd* and *Bsal*). This pool is referred to as NTC in the rest of the text. All concentrations given in this study are concentrations assessed in the samples that were calculated by taking into account the sample dilution in the ddPCR mix (i.e. 1 μl of eDNA sample with 21 μl of ddPCR mix).

GBlocks gene fragments specifically designed to be amplified by the specific primers were synthesized and used as positive controls (*Bd* 188 bp – 5′-GTTGTTTTTTCAAAAAACACCCTTGATATAATACAGTGTGCCATATGTCACGAGTCGAACAAAATTTATTTATTTTTTCGACAAATTAATTGGAAATGATTTTAATTTAATTGAAAAAAATTGAAAATAAATATTAAAACAACTTTTGACAACGGATCTCTTGGCTCTCGCAACGATGAAGAACGCAG-3′ and *Bsal* 152 bp – 5′-CTCAGTGAATCATCGAATCTTTGAACGCACATTGCACTCTACTTTGTAGAGTATGCCTGTTTGAGAATCAATAGTATTTTCTTGTTCTATTTTTCTTTTTTTAATTCATTTCCTTGTCTTTTTATATCATCTAAAAAGTGATATAAAAATAG-3′) respectively at 5.5^.^10^−6^ ng/μl and 6^.^10^−6^ ng/μl for *Bd* and *Bsal*.

### Double threshold approach as inhibition and artefact countermeasure

As described in [42], the inhibition level was specifically assessed in each pond for the two ddPCR assays. In short, for each pond, three positive control reactions (comprising 1 μl of gBlock gene fragments DNA described above) were respectively spiked with 1 μl eDNA extracted from three replicates. This allowed taking into account the impact of inhibition on the fluorescence level of positive amplification events for each pond [42]. In order to assess the fluorescence level in non-inhibited positive reactions for each of the two assays, 21 positive control replicates were generated with 1 μl gBlocks alone.

From raw fluorescence amplitude measurements and approximating their distribution as normal either for negative and positive amplification events, two correction factors were calculated for each pond: an Upper and a Lower Threshold Correction Factor (UTCF and LTCF [42]). These correction factors were then used to define an upper threshold (to sieve out the high fluorescence artefacts produced by ddPCR i.e. ‘stars’ [42]) and a lower threshold (to sieve out intermediate fluorescence amplifications between positive and negative amplification events i.e. ‘rain’ cf. Biorad documentation). The use of these two thresholds produced a consistent and specific delineation of positive amplification events in replicates processed from each pond. It allowed, in each sample, the positive amplification events delineation process to fit both the baseline fluorescence shifting and the fluorescence dropping due to the various inhibition level in ponds [42]. Through this process, absolute concentrations of the samples were recovered (Table S2).

### Limit of blank, limit of detection and limit of quantification

For both assays, several metrics were calculated based on NTC and a decimal dilution series of gBlock fragments (ranging from 5.5 ng/μl to 5.5^.^10^−10^ ng/μl and 6 ng/μl to 6^.^10^−10^ ng/μl for *Bd* and *Bsal* respectively, with 6 replicates for each concentration) [69]: 1) the Limit of Blank (LOB), defined as the highest concentration that can be found in NTC, was determined from 190 NTC for *Bd* and 182 NTC for *Bsal* (LOB = mean _NTC_ + 3 SD _NTC_); 2) the Limit of Detection (LOD), defined as the lowest concentration of target DNA that can be detected with sufficient confidence, was derived from LOB using the standard deviation from the lowest detectable concentration of positive control (LOD = LOB + 3 (SD_low concentration sample_)). To strongly minimize false positives, a 99.73% confidence interval was chosen for LOB and LOD calculations. The LOQ (Limit of Quantification) i.e. the lowest concentration that can be assessed in 90% of the replicates, was established from the decimal dilution series of gBlock fragments.

### Decision making in *Bd*/*Bsal* detection (Fig. 3)

In a first pass, the 10 field replicates from each pond were analyzed for the presence of *Bd* and *Bsal*. If at least one of the replicates exhibited a concentration higher than the LOD, the pathogen was considered as detected on the site. If no replicate yielded any signal, the site was considered negative for the targeted pathogen. If one or several of the field replicates produced a signal below LOD, these replicates were repeated 12–13 times. In addition, all the other replicates from the site were replicated two times in order to compensate for the sampling bias generated by the composite sampling method. If no signal over the LOD was obtained from these additional technical replicates, the frequency of the below-LOD positive amplification events for the whole site was tested against the frequency of false positives in NTC assessed for each assay (with a total of 30 + 12-13n replications per site, n being the number of < LOD signals obtained in the first pass; 43 replicates total for sites when *n* = 1 and 82 when *n* = 4 (Tables 1 and 2)). The intrinsic dependency of replicates from the composite sample collected in each site allowed this pooling of amplification results at site level for statistical analyses. The statistical comparison between the frequencies of false positives in NTC and below-LOD signals in the targeted sites allows the recovery of pathogen detection information with a higher level of confidence for signals that would have been otherwise discarded. The same process was applied to the results obtained from amphibian swabbing with the difference that the statistical test of below-LOD results was used at the specimen level.

### Statistics and data analysis

The test of the frequency deviation between artefacts in NTC and below-LOD amplifications with Pearson’s Chi-squared (with simulated *p*-values based on 2000 Monte Carlo simulation replicates) along with the calculation of the correction factors were conducted in the RStudio environment 2022.02.3 (Build 492) [70] with R version 4.2.0 [71]. Maps were generated using QGIS 3.2.1 [54].

### Supplementary Information

**Additional file 1.**

**Additional file 2.**

**Additional file 3.**

**Additional file 4.**

## Data Availability

Raw data and materials are available on request.
